# The Structure of Classical Swine Fever Virus N^pro^: A Novel Cysteine Autoprotease and Zinc-Binding Protein Involved in Subversion of Type I Interferon Induction

**DOI:** 10.1371/journal.ppat.1003704

**Published:** 2013-10-17

**Authors:** Keerthi Gottipati, Nicolas Ruggli, Markus Gerber, Jon-Duri Tratschin, Matthew Benning, Henry Bellamy, Kyung H. Choi

**Affiliations:** 1 Department of Biochemistry and Molecular Biology, Sealy Center for Structural Biology and Molecular Biophysics, The University of Texas Medical Branch at Galveston, Galveston, Texas, United States of America; 2 Institute of Virology and Immunology, Mittelhäusern, Switzerland; 3 Bruker AXS, Madison, Wisconsin, United States of America; 4 Center for Advanced Microstructures and Devices, Louisiana State University, Baton Rouge, Louisiana, United States of America; Institut Pasteur, France

## Abstract

Pestiviruses express their genome as a single polypeptide that is subsequently cleaved into individual proteins by host- and virus-encoded proteases. The pestivirus N-terminal protease (N^pro^) is a cysteine autoprotease that cleaves between its own C-terminus and the N-terminus of the core protein. Due to its unique sequence and catalytic site, it forms its own cysteine protease family C53. After self-cleavage, N^pro^ is no longer active as a protease. The released N^pro^ suppresses the induction of the host's type-I interferon-α/β (IFN-α/β) response. N^pro^ binds interferon regulatory factor-3 (IRF3), the key transcriptional activator of IFN-α/β genes, and promotes degradation of IRF3 by the proteasome, thus preventing induction of the IFN-α/β response to pestivirus infection. Here we report the crystal structures of pestivirus N^pro^. N^pro^ is structurally distinct from other known cysteine proteases and has a novel “clam shell” fold consisting of a protease domain and a zinc-binding domain. The unique fold of N^pro^ allows auto-catalysis at its C-terminus and subsequently conceals the cleavage site in the active site of the protease. Although many viruses interfere with type I IFN induction by targeting the IRF3 pathway, little information is available regarding structure or mechanism of action of viral proteins that interact with IRF3. The distribution of amino acids on the surface of N^pro^ involved in targeting IRF3 for proteasomal degradation provides insight into the nature of N^pro^'s interaction with IRF3. The structures thus establish the mechanism of auto-catalysis and subsequent auto-inhibition of *trans*-activity of N^pro^, and its role in subversion of host immune response.

## Author Summary

Mammalian cells respond to viral infection by inducing an innate immune response involving interferon-α/β that mediates cellular antiviral defenses. Viruses, in turn, have evolved mechanisms to counter the host's innate immune response by inhibiting the interferon response. Pestiviruses use the virally encoded N-terminal protease (N^pro^) to suppress interferon-α/β induction. N^pro^ first cleaves itself off from the viral polyprotein using its own cysteine protease activity. Released N^pro^ then interacts with interferon regulatory factor-3 (IRF3), a transcriptional activator of interferon-β, and induces proteasome-mediated degradation of IRF3. We have determined the crystal structure of N^pro^ from classical swine fever virus. N^pro^ has a unique protease fold consisting of two domains. The N-terminal domain carries the protease active site and has the C-terminus, the auto-cleavage site, bound in the active site. Thus, following auto-cleavage at the C-terminus, N^pro^ obstructs the catalytic site preventing further activity, making the protease active only once in its lifetime. The C-terminal domain carries a zinc-binding site that is required for interaction with IRF3. Surface mapping of the N^pro^ residues essential for subversion of interferon induction provides insight into the interaction with IRF3 and its subsequent degradation. To our knowledge, this is the first structure of a direct IRF3 antagonist.

## Introduction

Cells sense RNA virus infections by pattern recognition receptors (PRR) such as Toll-like receptors and the cytosolic RIG-I and MDA5 that recognize different forms of single-stranded and double-stranded viral RNA [1], [2]. Engagement of these PRR triggers a signaling cascade leading to phosphorylation and subsequent activation of the interferon regulatory factor-3 (IRF3). Activated IRF3 then translocates into the nucleus where it induces transcription of the interferon-α/β (IFN-α/β) genes. This activation is essential for the host to mount innate and adaptive anti-viral responses [3]. Viruses have evolved a multitude of strategies to counter the initial steps of the host's innate immune activation [4], [5]. IRF3 is targeted by many different viruses that use virus-encoded proteins to counteract IRF3 functions. A few examples of viral proteins targeting IRF3 are NSP1 of rotavirus, ICP0 of herpes virus, the leader protein of mengovirus and of Theiler's murine encephalomyelitis virus, the ML protein of Thogoto virus, and the NS1 and NS2 proteins of bovine and human respiratory syncytial virus [6], [7], [8], [9], [10], [11], [12], [13]. The mechanisms of IRF3 antagonism employed by these viral proteins vary, and include inhibition of phosphorylation, nuclear translocation, and assembly of the transcription complex, as well as targeting IRF3 for proteasomal degradation.

Pestiviruses, such as bovine viral diarrhea virus (BVDV) and classical swine fever virus (CSFV) use a virally encoded protein, the N-terminal protease (N^pro^), to suppress the transcriptional activation of the IFN-α/β genes by interacting with IRF3 and inducing its ubiquitination and proteasome-dependent degradation [14], [15], [16], [17], [18]. N^pro^ also interferes with the activity of IRF7 in plasmacytoid dendritic cells [19]. Unlike the other viral proteins encoded by the pestivirus genome, N^pro^ has no counterpart in the other members of the *Flaviviridae* family. The N^pro^ protein is a leader cysteine autoprotease that cleaves itself from the nascent polyprotein during translation of the viral mRNA, freeing itself for innate immune suppression activities. Self-cleavage of N^pro^ releases the core protein that becomes a structural component of the virion, probably by associating with the viral RNA genome and forming a nucleocapsid [20], [21], [22]. N^pro^ is not associated with virions and is dispensable for virus replication [23]. Interestingly, following the first self-cleavage reaction, N^pro^ does not possess any proteolytic *trans*-activity [24]. Sequence comparison with other known protease families showed that N^pro^ has a unique sequence and a novel arrangement of catalytic residues, and hence it is classified as its own C53 protease family [25]. The predicted catalytic triad of N^pro^ is Glu22, His49 and Cys69, which differs from the known catalytic triads in serine and cysteine proteases. For example, the catalytic triad in papain-like cysteine proteases is Cys25-His159-Asn175, and that in subtilisin-like serine proteases is Asp32-His62-Ser245. Additionally, the catalytic activity of N^pro^ is not inhibited by typical cysteine protease inhibitors such as antipain dihydrochloride and by the serine protease inhibitor aprotinin [21]. There is strong evidence that the function of N^pro^ in IRF3 degradation and inhibition of IFN-α/β induction is independent of its autoproteolytic activity, since mutation of the catalytic Cys69 to Ala had a minimal effect on inhibition of IFN-α/β induction by N^pro^ [26], [27]. However, mutation of His49 resulted in a loss of IFN-antagonistic activity, indicating that there is spatial overlap of regions that are involved in proteolysis and those responsible for IRF3 interactions [15], [16], [26]. We previously reported that N^pro^ has two domains, a catalytic N-terminal domain and a zinc-coordinating C-terminal domain, and that a zinc-binding TRASH motif in the C-terminal domain is required for binding IRF3 and subverting IFN-α/β induction [28].

Here, we report the crystal structures of CSFV N^pro^ and a C168A cleavage site N^pro^ mutant to 1.6 Å resolution. To our knowledge, this is the first structure of an IRF3 antagonist. N^pro^ has a unique ‘clam shell’-like protease fold that is distinct from other known proteins including all known proteases. As predicted, N^pro^ consists of two domains, a cysteine protease domain and a zinc-binding domain. The active site of N^pro^'s cysteine protease is formed by a catalytic dyad, Cys69 and His49. Contrary to previous reports, Glu22 is not in the active site. The C-terminus of the protein that constitutes the self-cleavage site is not only bound in the protease active site, but also contributes an integral β-strand to the central β-sheet that makes up the active site. Thus, the C-terminus of N^pro^ occludes the catalytic site following cleavage, inhibiting any *trans*-activity of the protease and limiting the activity of the enzyme to a single catalytic turnover. The C-terminal domain contains the zinc-binding TRASH motif that is indispensable for binding IRF3 and targeting it for proteasomal degradation. Taken together, the N^pro^ structures presented here establish the mechanism of auto-catalysis and subsequent auto-inhibition of N^pro^, and provide insight into its interaction with IRF3 in subversion of host innate immune responses.

## Results

### Structure determination of N^pro^

**Figure 1:**
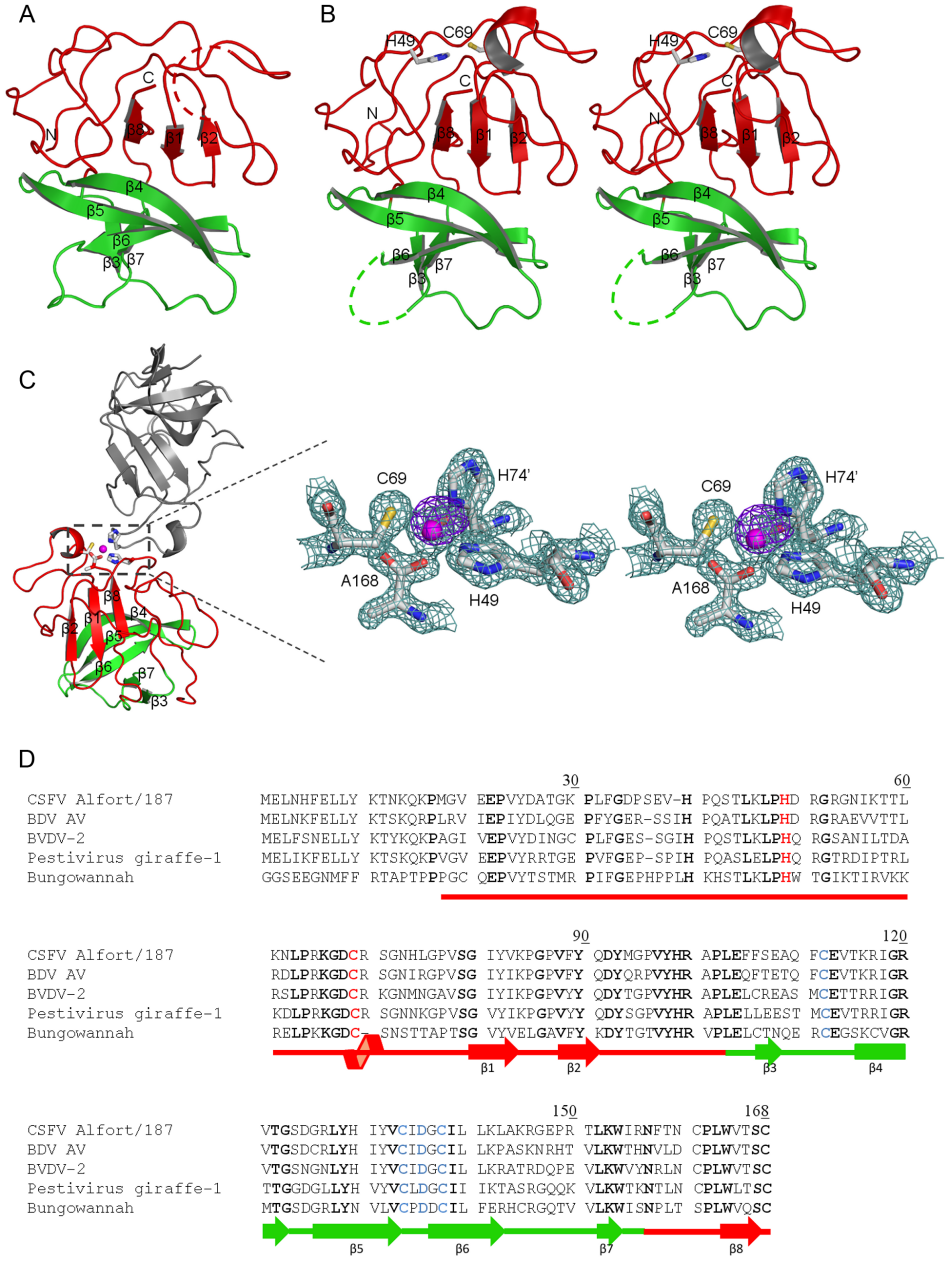
Crystal structure of CSFV N^pro^. (a) Ribbon representation of wild type and (b) Stereoview of the C168A N^pro^. The protease domain is shown in red and the zinc-binding domain in green. Disordered residues (65 to 71 in wild type and 145 to 149 in C168A mutant) are indicated by dashed lines. Secondary structural elements, as well as the N- and C-termini, are labeled. (c) Ribbon diagram of N^pro^-C168A mutant and its symmetry mate (grey) involved in zinc coordination at the catalytic site. Amino acids involved in the coordination complex (Cys69, His49, terminal carboxylate of Ala168 of one monomer and His74' from the neighboring monomer) are shown in sticks. Zinc atom is shown in magenta. Inset shows the close-up view of the zinc-binding site with the 2Fo-Fc electron density map contoured at 2σ (cyan). The anomalous difference Fourier map (blue) calculated with data collected at the zinc absorption edge (λ = 1.2823) is contoured at 8σ. (d) Sequence alignment of pestivirus N^pro^. The alignment includes the CSFV strain Alfort/187, Border Disease virus (BDV) strain AV, BVDV-2, Pestivirus giraffe-1, and the Bungowannah virus (GenBank accession numbers X87939.1, ABV54604.1, AAV69983.1, NP_777520.1, and DQ901403.1, respectively). Amino acid numbering corresponds to the CSFV Alfort/187 sequence. The conserved residues are in bold, the cysteine protease dyad is in red and the TRASH sequence motif is in blue. The domains are color coded as in (a). The secondary structure elements are indicated below the sequence. α-helix and β-strands are shown as coil and arrows, respectively.

Both crystal structures of N^pro^ were determined using deletion mutants that lack the first 17 amino acids (N^pro^-Δ17N). The first 19 amino acids are not essential for proteolytic activity of N^pro^ and can be deleted without affecting the *in vitro* protease activity [21], [26]. The 19 residue deletion also does not interfere with the ability of N^pro^ to block the IFN-α/β induction in cell culture [26]. Thus the structures represent biologically relevant forms of N^pro^. The C168A protein was expressed as N^pro^-Δ17N protein that contains a Cys168 to Ala mutation along with an additional four residues at the C-terminus, ^169^Ser-Asp-Asp-Gly^172^, a sequence that corresponds to the first four amino acids of the core protein. This construct was intended to trap a substrate-bound form of N^pro^; by mutating the cleavage-site residue, N^pro^ should not be able to catalyze self-cleavage at its C-terminus. Unexpectedly, the C168A mutant was active and the SDDG peptide at the C-terminus was cleaved, as confirmed by mass spectrometry (data not shown). Introduction of the Cys168 to Ala mutation resulted in different packing in the crystal lattice. The C168A N^pro^ crystals belong to the spacegroup P2_1_2_1_2_1_ with one molecule per asymmetric unit and a solvent content of 54%. In contrast, wild-type N^pro^ crystals were of spacegroup P2_1_2_1_2 also with a monomer in the asymmetric unit, but with a solvent content of 35% (**Table 1**). Although it is not entirely clear why the C168A mutation resulted in such a dramatic change in packing, new crystal contacts at the active site contributed to the stabilization of the helix carrying the nucleophilic Cys69, which was disordered and not visible in the native structure (**Figure 1a–b**). In the C168A structure, the side chains of Cys69, His49 and the C-terminal carboxyl group of one molecule, along with the side chain of His74 from a neighboring molecule, coordinated a zinc atom (**Figure 1c**). Since His74 is not conserved among pestiviral N^pro^ proteins, coordination of zinc near the active site is most likely a result of crystal packing interactions and may not be physiologically relevant. Presence of zinc was confirmed by determining the anomalous difference map from single wavelength anomalous dispersion (SAD) data collected at the zinc absorption edge. The distances between zinc and the coordinating groups are 1.9, 2.1, 2.1 and 2.3 Å for the carboxy-terminus, two histidines (His74' and His49), and cysteine (Cys69) residues, respectively, all of which are consistent with distances reported for tetrahedral zinc coordination [29]. The structure of the C168A mutant can be superimposed on the wild type structure with a root-mean square deviation (R.M.S.D.) of 0.38 Å for 136 Cα atoms. Thus, there is no gross structural difference between the wild-type and C168A mutant.

**Table 1:** Data collection, phasing and refinement statistics.

	N^pro^-Δ17N	N^pro^-Δ17N	N^pro^-Δ17N-C168A	N^pro^-Δ17N-C168A
	(Sulfur-SAD†)	(Native†)		(Zinc Anomalous)
**Data collection**				
Space group	P21212 (No. 18)	P21212 (No. 18)	P212121 (No. 19)	P212121 (No. 19)
Cell dimensions				
a, b, c (Å)	60.1, 65.4, 33.3	60.1, 65.4, 33.3	41.5, 58.3, 75.5	40.6, 58.3, 74.7
Wavelength (Å)	1.5418	1.5418	1.5418	1.282
Resolution (Å)	29.7 – 2.0	29.7 - 1.6	37.7 - 1.6	45.0 - 2.5
Rsym	0.035 (0.114)*	0.042 (0.318)	0.037 (0.452)	0.065 (0.196)
I/σI	14.48 (4.44)	12.6 (1.98)	40.6 (1.96)	24.4 (8.02)
Completeness (%)	99.7 (100.0)	99.9 (99.9)	99.2 (87.9)	100.0 (100.0)
Redundancy	51.6 (11.1)	34.3 (5.2)	5.3 (2.4)	5.5 (5.4)
**Refinement**				
Resolution (Å)	29.7 – 2.0	29.7-1.6	37.7-1.6	45.0 - 2.5
No. reflections	9424	17961	23012	12335
Rwork/Rfree		17.1/20.8	17.9/21.6	
No. atoms				
Protein		1121	1164	
Ligand/ion		0	16	
Water		224	262	
B-factors				
Protein		16.8	15.2	
Ligand/ion			36.8	
Water		28.6	26	
R.M.S. deviations				
Bond lengths (Å)		0.007	0.006	
Bond angles (°)		1.12	1.06	
Ramachandran Plot				
Most favored (%)		94.6	95.7	
Additional allowed (%)		5.4	4.3	
Disallowed (%)		0	0	

* Values in parentheses are for the highest-resolution shell.

† Data collected on the same crystal. Sulfur-SAD represents the high redundancy 2 Å data set. Native data includes the 2 Å data and additional high-resolution data.

### N^pro^ has a unique protease fold

N^pro^ is composed predominantly of β-sheets that adopt a unique ‘clam shell’-like fold. The protein can be divided into two distinct domains, the catalytic protease domain and the zinc-binding domain (**Figure 1**). The protease domain spans the N-terminus through residue 100 and also includes C-terminal residues 157 to 168. The domain harbors the protease active site along with the C-terminal protease cleavage site Cys168. The protease domain contains mostly coils without regular secondary structure and a single β-sheet formed by strands β1, β2, and β8. The first two β-strands of the sheet are contributed by the first 100 residues in the sequence. The last 6 residues at the C-terminus (163–168) form the final β-strand, and fold back into the protease active site, positioning the C-terminus Cys168 for cleavage (see the next section). The N^pro^ protease domain was predicted to be disordered, perhaps due to the abundance of proline residues; the domain contains twelve prolines corresponding to an average of one proline for every 7 residues. Most prolines are located in the loops on the surface of the protein, contributing to the unique fold of the protein. A search for similar folds using the DALI server resulted in zero instances, indicating that the catalytic domain of N^pro^ has a new fold. The zinc-binding domain of N^pro^ spans residues 101 through 156 and forms an anti-parallel β-sheet consisting of five β-strands, β3, β4, β5, β6, and β7 (**Figure 1**). This domain carries a conserved metal binding TRASH motif consisting of Cys112-Cys134-Asp136-Cys138 that coordinates a single zinc atom [28]. The TRASH motif is located at one end of the β-sheet. The interface between the protease and zinc-binding domains is mostly hydrophobic, and the C-terminal domain partially covers the final β-strand in the protease domain (see below).

### The active site of N^pro^ consists of the Cys69-His49 dyad, and does not include Glu22

**Figure 2:**
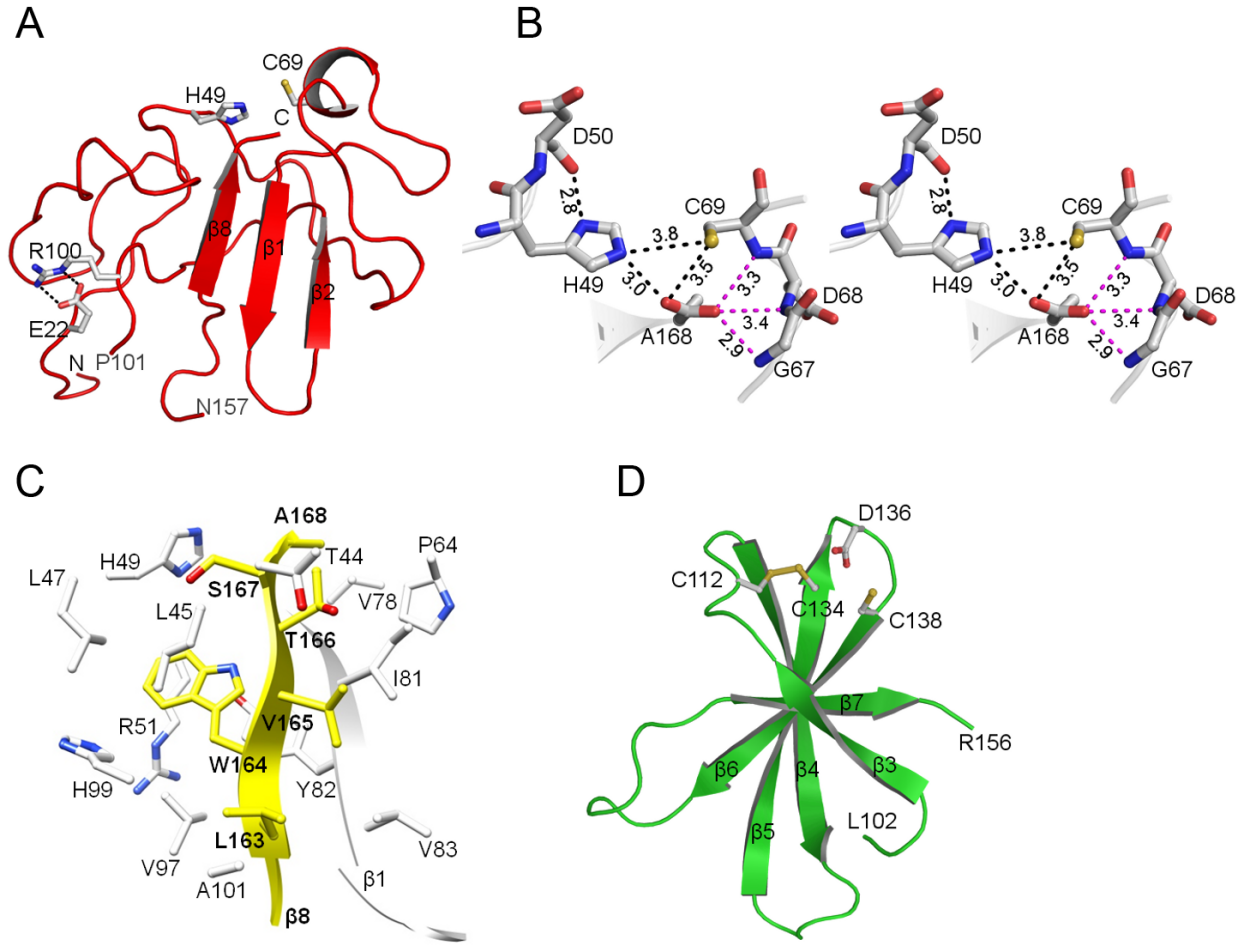
Structural domains of N^pro^. (a) The N-terminal protease domain of N^pro^. The catalytic dyad (Cys69 and His49) is shown in sticks. Glu22 originally predicted to be a part of the active site is at a distance of 23 Å from Cys69 and forms a salt bridge (dashed lines) with Arg100. (b) Stereo view of the active site of N^pro^. The catalytic dyad (Cys69 and His49) flanks the cleavage site Ala168. The distances in Å between the catalytic dyad and the terminal carboxylate in the active site are shown in black dashed lines. The hydrogen bonding distances between Ala168 and the oxyanion hole formed by the amide hydrogens of Gly67, Asp68 and Cys69 are also indicated (magenta dashed lines). (c) Substrate binding site of N^pro^. The C-terminal β-strand (β8), containing substrate sites P1 through P6, is shown in yellow, along with the surrounding residues forming the substrate binding site in the protease domain (white). The adjoining strand β1 that stabilizes β8 by main-chain hydrogen bonding interactions is also shown and labeled. (d) Zinc-binding domain of N^pro^. The TRASH motif (Cys112-Cys134-Asp136-Cys138) is shown in stick models on one end of the β-sheet. Zinc is missing in the structure and Cys112 and Cys134 form a disulfide bond, probably due to oxidizing conditions in the crystallization solution.

A cysteine protease triad in N^pro^ was predicted to include Glu22, His49 and Cys69 by site-directed mutagenesis in a cell-free translation system [21]. The active site of N^pro^ is solvent exposed on one end of the protease domain (**Figure 2a**). Cys69 is part of a single-turn helix formed by residues 68–71 in the C168A protein, while it is disordered in the native structure. Among the proposed protease triad, only Cys69, the nucleophile, and His49, the general base, are present to form a catalytic dyad flanking the C-terminal cleavage site. The sulfur atom of Cys69 is 3.8 Å away from the epsilon nitrogen of His49, and 3.5 Å away from the terminal carboxylate of Ala168. These distances are comparable to those seen in crystal structures of papain-like cysteine proteases, although the arrangement of the dyad (His49-Cys69) is different from that of papain (Cys25-His159) and other cysteine proteases. Since a zinc atom is present in the active site (figure 1C), this could lead to small artifacts in the observed active site geometry. Cysteine proteases often contain a stabilizing Asn/Asp residue in the vicinity of the catalytic His. No stabilizing anion group in the vicinity of His49 is apparent. However, the main chain carbonyl group of Asp50 is within a hydrogen-bonding distance of 2.8 Å of the delta nitrogen of His49, and thus could orient the imidazolium ring of His49 during catalysis (**Figure 2b**). Contrary to previous predictions, Glu22 does not complete a catalytic triad since its spatial location is approximately 23 Å from the nucleophilic Cys69 (**Figure 2a**). Instead, Glu22 forms a salt bridge with the conserved Arg100. Since mutation of Glu22, either a deletion or an Ala-substitution renders the protease inactive, breakdown of the salt bridge between Glu22 and Arg100 likely destabilizes the structure of the protease domain, resulting in the observed loss of proteolytic activity.

Both N^pro^ structures represent a product-bound form of the enzyme, providing the geometry of the catalytic residues around the scissile bond of Cys168. The C-terminal β-strand (β8) provides substrate specificity by positioning the C-terminal cleavage site residue 168 for hydrolysis near the protease dyad. The C-terminal carboxylate of residue 168, either Cys in the wild-type structure or Ala in the C168A mutant structure, has a clear density with planar geometry (**Figure 2b**). The terminal carboxylate forms hydrogen bonds with the main chain amides of Gly67, Asp68 and Cys69. These hydrogen bonding interactions would also help stabilize the tetrahedral intermediate during catalysis and thus form the oxyanion hole.

### Substrate specificity of N^pro^

N^pro^ cleaves the peptide bond between Cys168 and Ser169 in the viral polyprotein such that Ser169 then becomes the N-terminus of the core protein. The residues at the C-terminus of N^pro^ (the P sites) are reported to be essential for a functional N^pro^ protease, while the residues following the cleavage site (P' sites) can tolerate many amino acid substitutions [20], [24], [30]. The cleavage site for substrates is defined as …P3-P2-P1- P1'-P2'-P3'…, where a cleavage occurs between the P1 and P1' residues. The last seven C-terminal residues Pro^162^-Leu-Trp-Val-Thr-Ser-Cys^168^ (P7 to P1) form the final β-strand (β8) which is an integral part of a β-sheet. The β-strand is partially occluded by the C-terminal domain, such that the P7 to P4 site is not solvent accessible and enclosed in a hydrophobic environment. In addition to the typical main chain hydrogen bonding with β1, the β8-strand is stabilized by side-chain interactions, most notably hydrophobic interactions, involving Leu163, Trp164, and Val165. For example, Trp164 (P4) shown to be critical for protease activity is located in a hydrophobic environment consisting of the conserved residues Leu45, Leu47, Arg51, Tyr82, Val97, and His99 (**Figure 2c**). This is consistent with the highly conserved nature of the C-terminal residues and the mutational studies in which Trp164 to Ala substitution renders the protease inactive and prevents the release of N^pro^ from the BVDV-encoded polyprotein [20], [24]. The W164A mutation would likely destabilize the β-sheet and, by extension, the catalytic site of N^pro^ such that the enzyme is no longer able to carry out catalysis.

Cys168 is absolutely conserved in pestiviruses, and thus N^pro^ has been thought to be highly specific for Cys at the P1 site. Consistent with this prediction, Cys168 to Glu substitution abrogates the protease activity [30]. In our experiment, however, the recombinant C168A protein that contains the additional four residues (SDDG) from the core protein was proteolytically active, and the four C-terminal residues were cleaved by the protein. In the C168A structure, the side chain of Ala168 is located in a shallow hydrophobic pocket formed by Thr166, Pro64, Val78 and Gly80 (S1 subsite) (**Figure-2c**). The S1 subsite can only accommodate amino acids with small side chains. A long negatively charged Glu in the C168E protein thus would not fit in the subsite due to steric hindrance. Thus, although N^pro^ does not require Cys at the cleavage site, Cys168 is conserved in pestiviruses, suggesting that the residue may have an additional function other than participating in the protease catalysis.

### Mechanism of intramolecular product inhibition by N^pro^

Following auto-proteolysis at the C-terminus, the catalytic activity of N^pro^ is completely lost [24]. The structures presented here show that the C-terminal β-strand (one half of the product peptide) remains buried in the active site pocket, indicating that once cleaved, the C-terminus of N^pro^ acts as an intramolecular inhibitor and thus prevents *trans*-activity, i.e, the enzyme is inactive toward additional substrates. This is consistent with limited proteolysis results showing that the C-terminus of N^pro^ is protected from proteolytic degradation [28]. Additionally, the C-terminal β-strand (substrate) is also a part of the central β-sheet. Thus, no other peptide substrate can bind in the substrate binding site without disrupting the fold of the protease. In this way, N^pro^ has evolved to carry out only a single catalytic event.

An analogous autoprotease mechanism, viz., intramolecular product inhibition, has been reported for several proteins. For example, pestivirus NS2 is an autoprotease that cleaves its own C-terminus from the NS2-3 protein. Although the full-length NS2 is limited to a *cis*-cleavage reaction, deletion of at least four amino acids from the C-terminus was sufficient to allow the protein to cleave a substrate *in trans* [31], indicating that the C-terminal residues (substrate) do not participate in either the fold or the activity of the protease. In contrast, N^pro^ is unlikely to possess *trans*-cleavage activity even if the C-terminal residues are deleted from the protein because the C-terminal β-strand is critical for the fold and activity of the protease domain. Deletion of the C-terminal residues would result in an unstable protease rather than an active protease with an unoccupied substrate binding site. We have indeed observed that the deletion of the terminal 5 amino acids results in an insoluble protein, likely due to instability of the protein and the resultant formation of inclusion bodies upon expression in *E. coli* (unpublished data).

### Zinc-binding domain of N^pro^

The C-terminal zinc-binding domain (residues 101 to 156) forms an anti-parallel β-sheet consisting of five β-strands (β3, β4, β5, β6, and β7). Though the domain is not directly involved in the proteolytic mechanism, it serves as a structural scaffold for the N-terminal protease domain and shields the C-terminal β-strand. The C-terminal domain likely maintains the structural integrity of the protein until the final β-strand carrying the cleavage site (Cys168) is translated, which then enables the catalytic domain to acquire its active conformation, thus allowing cleavage of the peptide bond at the C-terminus of N^pro^. We have shown that this domain carries a conserved metal binding TRASH motif with the consensus sequence C-X_19–22_-C-X_3_-C (X being any amino acid) [28]. N^pro^ has a modified TRASH motif that consists of Cys112-Cys134-Asp136-Cys138, which coordinates a single zinc atom. Individual mutations of C112A/R, C134A, D136N, and C138A in the TRASH motif resulted in loss of zinc-binding, and also abolished IRF3 binding and subsequent inhibition of IFN-α/β induction when introduced into the virus. In the crystal structure, all four residues of the TRASH motif are located at one end of the β-sheet, consistent with previous biochemical data [28] (**Figure 2d**). The zinc-binding site consists of a loop that contributes the ligand Cys112 and a β-hairpin that contributes the other three ligands Cys134, Asp136, and Cys138, respectively. However, neither the wild-type nor the C168A structures contain a bound zinc atom at this site. Instead, a disulfide bridge was formed between Cys112 and Cys134. We surmise that the zinc atom escaped the binding site in the absence of a stable reducing agent in the crystallization conditions, which in turn allowed formation of a disulfide bridge. This displaced zinc atom could then be salvaged in the C168A protein, and coordinated by the secondary coordination site formed between two molecules of N^pro^ as a result of crystal packing (see above). The distance between Cys112 and Cys138, the furthest two residues in the proposed zinc-binding site, is 7.7 Å, which is too long to form a zinc coordination site. Since both residues are located in flexible loop regions, they may come closer upon zinc binding without the need for major conformational changes. Alternatively, one of the Cys residues may be required to maintain the geometry of the other residues in the zinc-binding site, and may not be directly involved in zinc binding. A water molecule could then occupy the fourth coordination site.

The TRASH motif was first described as a novel sequence motif for genes involved in copper homeostasis, and was predicted to have a treble clef fold [32]. The treble clef fold consists of a β-hairpin at the N-terminus and an α-helix at the C-terminus that contribute two ligands each for zinc-binding [33]. However, the zinc-binding site in N^pro^ does not resemble the treble clef fold or any other common zinc-finger motifs. It is close to a zinc ribbon in that the zinc-binding site contains a three-stranded anti-parallel β-sheet. Unlike a typical zinc ribbon that consists of two zinc knuckles (short β-strands connected by a turn) that each contribute two ligands, in N^pro^ one ligand comes from the loop connecting β3 and β4, and the other three from the strands β5 and β6 and the loop connecting them (Figure 2d). Since the zinc-binding residues in N^pro^ constitute a modified form of the TRASH motif, i.e., C-X_21_-C-X-D-X-C, it is not clear whether the zinc-binding motif in N^pro^ forms a subset of the TRASH motif or a new zinc coordinating sequence motif.

An intact zinc-binding site in N^pro^ is required for binding IRF3 and targeting it for proteasomal degradation in the host cell [28]. Similar to pestivirus N^pro^, rotavirus NSP1 and herpes virus ICP0 also inhibit IRF3 activation by binding to IRF3 and targeting it for proteasomal degradation [8], [34]. Both proteins also contain a conserved zinc-binding RING-finger motif (Cys_3_HisCys_4_) at their N-termini, and have been suggested to act as an E3 ubiquitin ligase. The E3 ligase transfers ubiquitin from the E2 conjugating enzyme to the substrate protein via direct interaction with the substrate protein. Although N^pro^ contains a zinc-binding motif, the structure of the zinc-binding site is rather different from the classical zinc-fingers and does not resemble the RING-finger motif, the typical fold of E3 ubiquitin ligase. Thus, it seems unlikely that N^pro^ functions as an E3 ubiquitin ligase and N^pro^ may regulate the IRF3 degradation via a mechanism different from that of rotavirus NSP1 and herpes virus ICP0.

### Interaction of N^pro^ with IRF3

The role of N^pro^ in the regulation of IRF3-dependent IFN-α/β induction is well-established in the pestiviral disease pathogenesis. Interaction of CSFV N^pro^ with IRF3 has been shown in cell-based and *in vitro* binding assays, and interaction between BVDV N^pro^ and IRF3 has been shown by immunoprecipitation, all of which were used to identify residues that affect its ability to interfere with IFN induction [15], [16], [26], [27], [28]. Mutations of the catalytic Cys69 had a minimal effect on IFN induction for both BVDV and CSFV N^pro^, indicating that the protease activity is not related to IRF3 binding [15], [26], [27]. However, mutations of His49 to Val or Leu resulted in a loss of IFN-antagonistic activity, suggesting at least partial structural overlap between the protease and anti-IFN functions of N^pro^ [15], [16], [26]. Point mutations of Glu22 to Leu or Val also abolished the anti-IFN activity of N^pro^ [15], [16], [26]. Cys112, Cys134, Asp136 and Cys138 in the zinc-binding domain were also required for the anti-IFN activity, as described previously [28]. N-terminal mutations of BVDV and CSFV N^pro^ have different consequences on the suppression of IFN-α/β production in infected cells [15], [16], [26]. In BVDV N^pro^ Leu8 to Pro substitution impaired its IFN-α/β antagonistic function. Although the mutant displayed binding to IRF3, it could no longer promote its ubiquitination and proteasomal degradation [16]. In contrast, CSFV N^pro^ that contains a deletion of the N-terminal 19 amino acids maintained its ability to inhibit IFN-α/β response [26]. Deletion of 24 or more amino acids at the C-terminus of N^pro^ also abolished the anti-IFN activity of N^pro^ [16], [26], [27].

**Figure 3:**
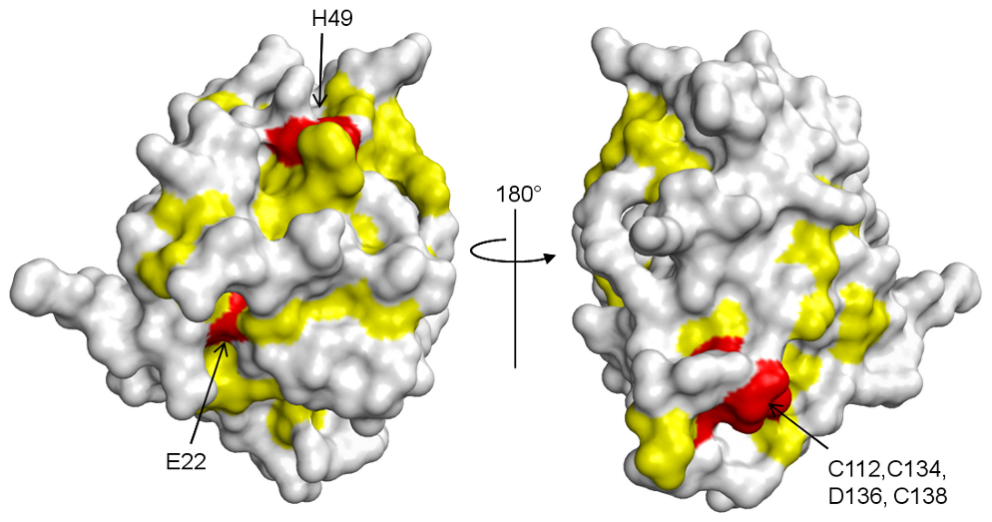
Spatial distribution of the residues involved in N^pro^-mediated proteasomal degradation of IRF3. The residues that are essential for the N^pro^-mediated IRF3 degradation (red), along with the conserved residues (yellow, bold letters in Fig. 1) are mapped onto the surface of N^pro^. The residues localize to two protein surfaces, one in each domain. The protease domain surface cluster (left) includes residues Glu22 and His49, and the zinc-binding domain cluster (right) is formed by the residues in the TRASH motif.

To determine if the residues involved in the anti-IFN response form a localized IRF3-binding surface, we mapped the above mentioned residues on the 3D structure of N^pro^, along with the conserved residues (**Figure 3**). Large deletions of the N-terminal 19–22 amino acids or the C-terminal 24 amino acids were not included in the surface mapping, since their loss of function may be caused by the disruption of protein folding. The residues form two spatial clusters on the opposite sides of the protein surface; one cluster is on a face of the protease domain, and the other on the zinc-binding domain (**Figure 3**). Whether IRF3 could simultaneously interact with both surfaces is not known. However, since the mutation on the N-terminus of BVDV N^pro^ (L8P) only disrupted ubiquitination of IRF3 and not its binding, the two surface clusters of conserved residues in each domain may account for different functions in anti-IFN activity; one for direct interaction with IRF3 and the other for interaction with cellular proteins in a downstream response leading to ubiquitination and degradation of IRF3 [16]. Based on previous experimental data and the surface distribution of residues involved in IRF3 binding, we speculate that the C-terminal Zn binding domain interacts with IRF3, whereas the protease domain would bind to a cellular protein involved in the ubiquitination reaction.

## Discussion

Cysteine proteases fall into one of two major groups, or clans based on their structural homology and evolutionary relationship. Clan PA proteases are evolutionarily related to the chymotrypsin family and have the common double β-barrel fold with catalytic residues located between the two β-barrels. The catalytic nucleophile can be either serine or cysteine arranged similar to His57-Asp102-Ser195 in the chymotrypsin sequence. The other clan, CA, comprises all papain-like cysteine proteases which consist of an N-terminal α-helical domain and the C-terminal β-barrel domain with the active site located in the cleft between the two domains. The arrangement of the catalytic residues in papain is Cys25-His159-(Asn175); Asn helps to orient the imidazolium ring of the catalytic His [35]. N^pro^ does not share sequence homology with any other known proteases, and thus was assigned to its own family of cysteine proteases, C53 [25]. The newly established catalytic His49-Cys69 dyad does not align in either sequence or structure with either type of cysteine protease, and the unique fold of the protein reported here supports this classification. One of the unique features of the protease is that the C-terminal residues, the substrate of its own protease activity, form a β-strand that contributes to the overall fold of the protein. This β-strand is further blocked by the zinc-binding domain, and the substrate binding site is partially enclosed in a hydrophobic environment. Such an arrangement of substrate peptide would prevent further access of any other endogenous substrates to the active site for cleavage, indicating that N^pro^ evolved to catalyze a single cleavage event. After self-cleavage, the product peptide remains bound in the active site pocket, making the protease permanently inhibited by its own C-terminus. In fact, the β-strand would not be able to be released without distorting the structure, resulting in the loss of protease activity. Additionally, the short α-helix containing the catalytic Cys69 is disordered in the wild-type N^pro^ structure, which may be an additional measure to deactivate the protease function after initial cleavage.

While our manuscript was under review, BVDV N^pro^ structures that lack the first 21 amino acids have been published [36]. The overall fold is similar to the fold presented here, and BVDV N^pro^ (PDB 3zfr) can be superimposed with an rmsd of 0.86 Å for the common 134 Cα atoms with CSFV N^pro^. The greatest deviations between CSFV and BVDV N^pro^ and among BVDV N^pro^s lie in the short helix containing the catalytic Cys69 and the loop preceding the helix (residues 63–69). This also contributes to two major differences in the structures and in their interpretation. First, the catalytic Cys69 and Cys168 forms a disulfide bond in BVDV N^pro^. Since this is an inactive state of the enzyme, an active form of the enzyme with reduced Cys69 was proposed to be in equilibrium with the non-productive form. Second, a bound hydroxide ion near Gly67 amide was proposed to deprotonate the catalytic Cys69 sulfhydryl for the nucleophilic attack. His49 would then function as oxyanion hole and polarize the scissile bond, instead of forming an imidazolium (His49)-thiolate (Cys69) ion pair. In comparison, CSFV N^pro^ structures do not have a disulfide bond between Cys69 and Cys168 because the active site-containing helix is disordered in the wild-type N^pro^ and Cys168 is replaced in the C168A N^pro^. Since C168A is as active as the wild-type N^pro^ in our hands, disulfide bond formation is not a required step in catalysis. In addition, no water molecule was observed near the Gly67 amide. The Gly67 amide, along with Asp68 and Cys69 amides, is within hydrogen-bonding distance to the C-terminal carboxylate (Figure 2). Thus, the CSFV N^pro^ structure supports the classical cysteine protease mechanism rather than the catalytic mechanism proposed for BVDV N^pro^. Although differences in crystallization conditions and protein preparations (native vs refolding) could have led to the observed differences in the catalytic site, it seems unlikely that BVDV and CSFV N^pro^ utilize a different mechanism to catalyze the cleavage reaction. Since both proteins could have a distorted active site geometry either from the disulfide bond formation between Cys69 and Cys168 in BVDV N^pro^ or zinc coordination in CSFV N^pro^, the measurement of pKa of the Cys69 and His49 may be required to distinguish between the two mechanisms.

The release of N^pro^ from the polyprotein subsequently sets the stage for suppression of innate immune responses. Pestivirus N^pro^ binds and degrades IRF3 via ubiquitination and the proteasomal degradation pathway, and thus subverts the IFN-α/β induction in host cells [16], [17], [18], [27]. However, following the initial binding to IRF3, the mechanism of IRF3 degradation is still unknown. In addition, although many residues have been indicated to be involved in IRF3 binding and subsequent IFN subversion, it is not known whether the mutations directly affect IRF3 degradation or simply decrease protein stability. For example, Glu22 was proposed to be important for both proteolytic activity and IFN subversion functions of N^pro^ [15], [16], [21], [26]. In light of the crystal structures presented here, it is likely that the mutation would destabilize the protein folding leading to loss of function. Nonetheless, the N^pro^ residues involved in the anti-IFN function cluster into two patches on opposite sides of the protein surface (**Figure 3**), suggesting there might be distinct functions for each of the patches. Since N^pro^ is unlikely to ubiquitinate IRF3 directly, other cellular proteins probably need to bind to the N^pro^-IRF3 complex for ubiqutination and degradation to occur. This could also explain the observations that the N-terminal mutations of CSFV and BVDV N^pro^ have different consequences in subversion of interferon induction. Although the same cellular proteins are likely recruited to CSFV and BVDV N^pro^, specific residues involved in the interaction between N^pro^ and the protein partner may be different.

Several N^pro^-binding proteins including IRF-7, HAX-1, I*κ*Bα, and TRIM56 have been identified [19], [37], [38], [39]. IRF7 is a transcription factor for interferon-α genes and induced by type I interferon. N^pro^ also interferes with the function of IRF7 in pDC and thus dampens interferon-α induction during viral infection. Similar to IRF3 and N^pro^ interactions, the IRF7 interactions with N^pro^ rely on the zinc-binding domain of N^pro^. In particular, individual mutations of TRASH motif residues (C112R, C112A, C134A, D136N, and C138A) in N^pro^ abolished the IRF7 interaction in mammalian two-hybrid assays [19]. However, interaction between IRF7 and N^pro^ does not induce proteosomal degradation of IRF7, suggesting a different mechanism of N^pro^-mediated IRF7 antagonism. The significance of N^pro^ interactions with HAX-1, I*κ*Bα, and TRIM56 in viral pathogenesis is less clear. HAX-1 and I*κ*Bα are involved in controlling cell survival, while TRIM56 is involved in antiviral response. Interestingly, the consensus sequence for HAX-1 binding site was suggested to be present in N^pro^ between residues 110 and 135. The peptide corresponding to N^pro^ residues 106–143 interact with HAX-1 in co-precipitation assays [37]. This N^pro^ peptide contains an intact TRASH motif, and it is likely that HAX-1 binds to the zinc-binding surface as with IRF3 and IRF7. Several additional proteins interacting with N^pro^ were identified in random screens, but the functional relevance of these interactions have not yet been characterized [39], [40]. Identification of cellular proteins that interact with N^pro^ and their interaction studies are essential next steps towards understanding how binding of N^pro^ to IRF3 leads to the degradation of IRF3 and N^pro^-mediated viral pathogenesis.

## Materials and Methods

### Plasmid constructs

The N^pro^ gene of CSFV strain vA187-1 (Alfort/187, GenBank accession number X87939) [41] was amplified by PCR from pA187-1 and cloned into pCR4-TOPO (Invitrogen). The DNA fragment containing the N^pro^ gene was then subcloned into the NdeI and XhoI restriction sites of pET-15b vector to obtain pET-6H-throm-N^pro^(Alf) [28]. The N-terminal deletion mutants were designed based on limited proteolysis results [28]. The N^pro^ construct lacking the first 17 amino acids (N^pro^-Δ17N) was amplified from the full-length construct using the oligonucleotide primers 5′ ggcagccatatgggagtggaggaaccggtatac 3′ (forward) and 5′ cggatcctcgagttagcaactggtaacccacaatgg 3′ (reverse). The PCR product was again sub-cloned into the pET15b expression vector between the NdeI and XhoI restriction sites. The C168A mutant with the additional four residues of the core protein (N^pro^Δ17N-C168A-SDDG) was cloned similarly using the reverse primer 5′ gtggtgctcgagttagccatcatcagaggcactggtaac 3′. All constructs were verified using DNA sequencing. The resulting proteins have a hexa-histidine tag (His-tag) and the thrombin cleavage sequence (LVPRGS) on the N-terminus of the protein.

### Expression and purification of N^pro^ proteins

The N^pro^ mutants were expressed and purified as described in ref. 28. His-tagged N^pro^ proteins, purified using Talon metal affinity chromatography resin (Clontech) were pooled and dialyzed in buffer A (20 mM Tris pH 8.0, 100 mM NaCl and 5 mM β-mercaptoethanol) overnight, and the N-terminal His-tag cleaved using thrombin protease immobilized on agarose beads (ThermoScientific). The cleavage reaction was performed with 1% thrombin (w/w) at room temperature in buffer A for 4 hrs. Cleaved N^pro^-Δ17N was separated by passing the mixture through the Talon resin equilibrated in buffer A. N^pro^-Δ17N-C168A was purified similarly except that 10% glycerol was added during thrombin cleavage of the His-tag. Glycerol was necessary to stabilize the protein during the cleavage reaction at room temperature. The protein was >95% pure judged by SDS-PAGE. Both proteins were monomers in solution as judged by size-exclusion chromatography.

### Crystallization of N^pro^-Δ17N proteins

Proteins were concentrated to ∼4.5 mg/ml. Initial crystallization trials were conducted using the sitting drop vapor diffusion method in 96-well plates using a Phoenix RE liquid handling robot (Rigaku). 200 nL protein solution was mixed with equal volumes of a range of precipitants obtained from commercially available crystal screens. Crystals appeared in several conditions within a week. Diffraction quality crystals of N^pro^-Δ17N grew in 25% PEG3350, 0.2 M MgCl_2_ (or 0.2 M (NH_4_)_2_SO_4_) and 0.1 M Hepes pH 7.4. N^pro^-Δ17N-C168A crystallized in 25% PEG3350 0.2 M (NH_4_)_2_SO_4_ (or 0.2 M Li_2_SO_4_) and 0.1 M Hepes pH 7.5.

### Data collection, structure determination and refinement

The N^pro^-Δ17N crystals were cryo-cooled at 100 K using paratone as cryo-protectant. High redundancy data was collected using Bruker's Microstar microfocus X-ray Source equipped with a Platinum^135^ CCD detector. The data were indexed and merged using the Bruker AXS PROTEUM2 software suite for X-ray crystallography (Bruker AXS (2010). PROTEUM2, Version 2010.5, Bruker AXS Inc., Madison, Wisconsin, USA). The crystals diffracted to 1.6 Å resolution and belonged to the space group P2_1_2_1_2 with a = 60.1, b = 62.6, c = 30.8 Å. The solvent content was 35% with a monomer in the asymmetric unit. The structure of N^pro^-Δ17N was solved via the single wavelength anomalous dispersion method (SAD) using the anomalous signal present in sulfur atoms illuminated by a copper K-α home X-ray source. Determination of the positions of sulfur atoms, phasing, and calculation of electron density maps were performed using AutoSol wizard in the Phenix package [42], [43]. The initial atomic model was obtained using the Autobuild wizard in Phenix [44]. The final model was achieved using manual model building with the program O [45] followed by iterative cycles of refinement with phenix.refine. All residues from Glu21 to Cys168 were visible in the electron density map except residues 65–71, which encompasses the catalytic Cys69.

Diffraction data for N^pro^-Δ17N-C168A crystals was collected to 1.6 Å using a Rigaku FRE++ X-ray source and an RAXIS-IV™ detector at UTMB. Data was indexed in the space group P2_1_2_1_2_1_ with unit cell dimensions of a = 41.5, b = 58.3, and c = 75.5 Å, different from the N^pro^-Δ17N protein. The solvent content of the crystals was 54%. The structure of N^pro^-Δ17N-C168A was determined using molecular replacement with the N^pro^-Δ17N structure as a model. An atomic model was built using Auto-Build, and phenix.refine was used for refinement of the final model. All residues from Met18 to Cys168 were visible except amino acids 145–149. New crystal packing interactions involving the active site contributed to the stabilization of the helix carrying the nucleophilic Cys69. Strong density (visible at >8σ) at the center of the coordination complex at the active site indicated the presence of a metal ion at the site. SAD data at the zinc absorption edge were collected at the Center for Advanced Microstructures and Devices (CAMD) synchrotron macromolecular crystallography beamline at Louisiana State University. An anomalous difference Fourier map was calculated to confirm the presence of zinc. Ramachandran plots for both structures were generated using the program PROCHECK [46] in CCP4. Data collection and refinement statistics are given in Table 1.

### Protein structure accession codes

The coordinates and associated structure factors for this publication have been deposited into the Protein Data Bank and assigned the following accession codes: 4H9J and 4H9K for the N^pro^-Δ17N and N^pro^-Δ17N-C168A, respectively.
